# Reducing Emergency Prescriptions (FP10s) Requiring Electronic Shared Care Agreement (ESCA) by North Hub Community Mental Health Team (CMHT), Birmingham & Solihull Mental Health Foundation Trust (BSMHFT)

**DOI:** 10.1192/bjo.2024.346

**Published:** 2024-08-01

**Authors:** Alisha Bakshi, Zora Bell, Matthew Stafford, Kate Jennings-Cole, Sanna Ceesay

**Affiliations:** Birmingham and Solihull Mental Health Trust, Birmingham, United Kingdom

## Abstract

**Aims:**

The community mental health team (CMHT) is actively involved in reviewing mental health patients who require commencing psychotropic medications. The responsibility to prescribe the psychotropic medications falls on the CMHT for the first 3 months. After this period, if the patient's mental health is stable, the prescribing role can be transferred to the GP by completion of an electronic shared care agreement (ESCA).

This project aimed to improve the management of emergency prescriptions (FP10s) requiring ESCA within the North Hub CMHT, BSMHFT focussing on reducing administrative time in receiving numerous urgent phone calls for repeat prescriptions, timely completion of ESCA and updating the electronic prescribing system.

**Methods:**

Data collection was done by logging the numbers of the following on a weekly basis:

1. FP10s issued.
2. Calls related to FP10s.
3. ESCA sent.

Baseline data was collected over 11 weeks to analyse practice. Plan-do-study-act (PDSA) cycle was used to improve the processes from January to August 2023. Identified PDSA cycles included:

1. Clinician prompt reminders to check ESCA status.
2. Document FP10s instances on issue and inform patient about ESCA during outpatient appointments.
3. A 4-week system for managing FP10s at reception desk.
4. Increase consistent use of and access to EPMA.

Data was collected again for 4 weeks in December 2023 to assess sustainability of the implemented changes.

**Results:**

This project resulted in a 14% reduction in the number of FP10s requiring ESCA and a 27% reduction in the number of calls for FP10s from January to August 2023. Data measuring sustainability in December 2023 showed a total reduction of 64% from the baseline of 28 FP10s per week at the beginning of the project (January 2023) to an average of 10 FP10s issued per week in December 2023.

**Conclusion:**

In conclusion, patients benefit from having a clear understanding of where their medications will be issued from thus improving their experience with the mental health service. Having effective processes in the CMHT enables medical professionals to complete the ESCA in a timely manner. Altogether this reduces burden on all professionals and reduces costs of prescribing by transferring the prescribing responsibilities to GPs. This project has been effective in reducing the number of weekly emergency FP10s issued. The 4-week system of managing FP10s at reception has now been included in the Medication Management's new procedure and guidance and is being introduced across all CMHTs in BSMHFT.

